# Association between order of eating and thinness among preschool children in Taizhou, China: a cross-sectional study

**DOI:** 10.3389/fped.2025.1654992

**Published:** 2025-08-21

**Authors:** Jingyun Yang, Yao Huang, Huilan Wu, Yixin Wu, Yun Wang, Hailing Fan, Mengjia Chen, Lizhen Wang, Meixian Zhang

**Affiliations:** ^1^ Department of Pediatrics, Taizhou Hospital of Zhejiang Province Affiliated to Wenzhou Medical University, Linhai, Zhejiang, China; ^2^ Department of Pediatrics, The Second People’s Hospital, Luqiao, Zhejiang, China; ^3^ Department of Pediatrics, Taizhou Hospital of Zhejiang Province Affiliated to Wenzhou Medical University, Taizhou Enze Medical Center (Group), Enze Hospital, Taizhou, Zhejiang, China; ^4^ Evidence-Based Medicine Center, Taizhou Hospital of Zhejiang Province Affiliated to Wenzhou Medical University, Linhai, Zhejiang, China

**Keywords:** order of eating, child, preschool, thinness, vegetables

## Abstract

**Background:**

Order of eating is reportedly associated with childhood obesity. However, few studies have examined the relationship between the order of consumption of vegetables and meat/fish and childhood thinness. We aimed to investigate the effect of the order of consumption of meat/fish and vegetables on the risk of thinness in preschool children.

**Methods:**

From December 1, 2021, to January 31, 2022, 419 thinness and 1,204 normal-weight preschool children were selected from kindergartens in Taizhou, China. We used a questionnaire to determine whether the children ate vegetables or meat/fish first at mealtimes and analyzed the association between the first food consumed and children's thinness status.

**Results:**

Overall, 53.4% and 46.6% of the children ate vegetables and meat/fish first, respectively. The percentage of eating vegetables first was higher in children with thinness than that in normal weight (58.95% vs. 51.41%, *P* = 0.008). After adjusting for sex, parental education and parental BMI category, the odds ratio for being thin was 1.280 for children who ate vegetables first (95% confidence interval: 1.016–1.612, *P* = 0.036) compared with those who ate meat/fish first.

**Conclusions:**

Our study revealed that order of eating was associated with childhood thinness among preschool children. For children with thinness, we do not recommend that they eat vegetables first, but rather encourage them to eat meat/fish first.

## 1 Introduction

Undernutrition adversely affects the growth and development of children and the severity of underdevelopment is determined by the degree of undernutrition. Furthermore, undernutrition in childhood, especially in the early years, is a major risk factor for impaired growth and weakened immunity (1, 2). Weight status is an effective indicator of undernutrition (3). Excessively low and high body weights, both, have an adverse effect human health. At present, there are many studies on the prevalence of childhood obesity; however, studies regarding childhood thinness are limited. According to the 2020 global data, 45 million children aged <5 years were affected by thinness (4). The prevalence of thinness among boys and girls aged 3–12 years in Shanghai, China was 13.92% and 18.45%, respectively (5). thinness markedly affects children's growth, development, and health, and its effect can persist into adulthood (6). In children, inhibited growth and development can lead to a weakened or impaired immune system and predisposition to infection (7). Additionally, poor nutrition can lead to weak bones and a relative lack of strength during exercise (7).

Furthermore, being thin is closely related to genetics and the external environment. An unbalanced diet and poor eating habits are the most common causes of childhood thinness. Previous studies have found that eating order is associated with the risk of obesity (8, 9), suggesting that eating order may affect weight through multiple pathways. Order of eating may affect weight through a variety of mechanisms. Vegetables are rich in dietary fibre and water, and have a low energy density, which can increase satiety more quickly and reduce subsequent intake of high-energy foods. This may lead to a reduction in overall calorie intake, thereby controlling body weight. In addition, eating vegetables first may slow down the absorption of carbohydrates, stabilise blood sugar levels, and reduce insulin fluctuations, thereby maintaining a more stable metabolic state (10–12).

The order of food consumption at the beginning of a meal varies across people. In China, a hot meals include vegetables, meat, fish, or soup. Chinese families often eat hot food together. The order of food consumption may affect children's weight status due to changes in their metabolic rate and the digestibility of any food consumed postprandially. Previous studies have found that the order of eating is associated with childhood obesity (13). However, only a few studies have examined the relationship between the order of consumption of vegetables and meat/fish and childhood thinness.

There are concerns about growth and physical development during the early stages of childhood. A nutritious and balanced diet is key to healthy development in children. Studies have found that the majority of a child's eating habits are formed during the preschool years (14), and early education at this age helps develop healthy eating patterns. Given this context, this study aimed at exploring the association between the order of consumption of vegetables and meat/fish and childhood thinness.

## 2 Methods

### 2.1 Study design and population

The study was conducted in Taizhou, which is a coastal city in east mainland China. From December 1, 2021, to January 31, 2022, 419 thinness and 1,204 normal-weight preschool children from local kindergartens were selected to participate in the study. An online survey was conducted using the Wen-Juan-Xing platform, which allowed the distribution of the questionnaire via WeChat (9, 15). The use of this platform allowed respondents to complete the questionnaire online and ensured wide accessibility to a large population in China. The parents of the preschool children included in the study completed the questionnaire. Voluntary participation in the survey was considered as the receipt of informed consent from both the children and their parents. This study was approved by the Ethics Committee of Taizhou Hospital, Zhejiang Province (approval number: K20220123). All the procedures were performed according to the guidelines of the institutional ethics committee. The participants' information was anonymized and maintained.

### 2.2 Weight statuses of the children and parents

Children's parents reported their own height and weight as well as that of their children. Body mass index (BMI) was calculated based on height (in meters) and weight (in kilograms). In accordance with the recommendations of the International Obesity Task Force, each child's BMI was adjusted for age and sex and converted to a standardized z-score (16). The children were categorized as thinness or normal weight, accordingly. Furthermore, the parents were divided into the following groups based on international weight cut-off values: thinness (BMI < 18.5 kg/m^2^), normal weight (18.5 kg/m^2^ ≤ BMI < 25 kg/m^2^), and overweight/obesity (BMI ≥ 30 kg/m^2^) (17).

### 2.3 Order of eating and other variables

To understand the children's order of eating, the following question was asked: “Does your child usually eat vegetables or meat/fish first at the beginning of a meal?” The two answer options provided were “vegetables before meat/fish” or “meat/fish before vegetables.” The information collected using the questionnaire included the child's sex, date of birth, residential area (urban or rural), parents' education level (junior or below, senior, university or above), occupation (brain work, physical work, or other), and annual family income (<120,000, 120,000–500,000, or >500,000 Chinese Yuan). The parents were also asked the following question: “How many brothers and sisters does your child have?” The responses available to them were 0, 1, and ≥2.

### 2.4 Statistical analysis

Categorical variables, including the children's demographic, parental, and family characteristics, were expressed as counts and percentages. Chi-square tests were used to assess the relationship between the order of vegetable and meat/fish consumption and the children's body weight status. Continuous variables, including the parents' ages, were expressed as mean ± standard deviation (SD) values. A *t*-test was used to evaluate the difference between children's body weight status and continuous variables. Logistic regression analysis was used to evaluate the association between the food consumed first at mealtimes and the thinness status of the children, in order to calculate the 95% adjusted odds ratio (OR) and confidence interval (CI). The following model sequence was constructed: Model 1 was adjusted for children's sex; Model 2 was additionally adjusted for mother's and father's educational levels; and Model 3 was additionally adjusted for the BMI categories of mothers and fathers. All the data were statistically analyzed using IBM SPSS software (version 26.0, SPSS Inc.). *P* < 0.05 was considered statistically different.

## 3 Results

### 3.1 Headings

Table 1 presents the basic characteristics of the participants. We analyzed 1,623 children aged 3–6 years, including 870 boys and 753 girls. The proportion of mothers who graduated from university or achieved higher academic degrees was lower for children with thinness than that for normal-weight (58.2% vs. 67.9%, *P* = 0.002). A higher proportion of mothers were thinness among the thinness children group as compared to the normal-weight children group (15.0% vs. 9.3%, *P* = 0.002).

**Table 1:** Comparison of basic characteristics between thinness and normal weight children.

Variables	Total (*n* = 1,623)	Body weight status		*P*
		Thinness (n* =* 419, 25.8%)	Normal weight (n* =* 1,204, 74.2%)	
Child-related characteristics				
Sex				0.387
Boy	870 (53.6)	217 (51.8)	653 (54.2)	
Girl	753 (46.4)	202 (48.2)	551 (45.8)	
Age (years)				0.470
3	404 (24.9)	111 (26.5)	293 (24.3)	
4	548 (33.8)	148 (35.3)	400 (33.2)	
5	516 (31.8)	121 (28.9)	395 (32.8)	
6	155 (9.5)	39 (9.3)	116 (9.6)	
Parental-related characteristics				
Father's age (years)	36.926 ± 9.545	37.549 ± 15.735	36.709 ± 6.051	0.121
Mother's age (years)	35.096 ± 6.678	34.926 ± 5.651	35.155 ± 7.002	0.545
Father's education level				0.104
Junior or below	236 (14.5)	74 (17.7)	162 (13.5)	
Senior	445 (27.4)	113 (27.0)	332 (27.6)	
University or above	942 (58.1)	232 (55.4)	710 (59.0)	
Mother's education level				**0**.**002**
Junior or below	205 (12.6)	63 (15.0)	142 (11.8)	
Senior	357 (22.0)	112 (26.7)	245 (20.3)	
University or above	1,061 (65.4)	244 (58.2)	817 (67.9)	
Father's occupations				0.444
Brain work	1,349 (83.1)	340 (81.1)	1,009 (83.8)	
Physical work	100 (6.2)	28 (6.7)	72 (6.0)	
Other occupation	174 (10.7)	51 (12.2)	123 (10.2)	
Mother's occupations				0.511
Brain work	1,244 (76.7)	317 (75.7)	927 (77.0)	
Physical work	80 (4.9)	18 (4.3)	62 (5.1)	
Other occupation	299 (18.4)	84 (20.0)	215 (17.9)	
Father's BMI category				0.057
Thinness	36 (2.2)	14 (3.3)	22 (1.8)	
Normal weight	919 (56.6)	248 (59.2)	671 (55.7)	
Overweight/Obesity	668 (41.2)	157 (37.5)	511 (42.4)	
Mother's BMI category				**0**.**002**
Thinness	175 (10.8)	63 (15.0)	112 (9.3)	
Normal weight	1,213 (74.7)	290 (69.2)	923 (76.7)	
Overweight/Obesity	235 (14.5)	66 (15.8)	169 (14.0)	
Family-related characteristics				
Residence				0.066
Urban	1,063 (65.5)	259 (61.8)	804 (66.8)	
Rural	560 (34.5)	160 (38.2)	400 (33.2)	
One-child family				0.063
Yes	702 (43.3)	165 (39.4)	537 (44.6)	
No	921 (56.7)	254 (60.6)	667 (55.4)	
Annual household income (CNY)				0.474
<120,000	320 (19.7)	89 (21.2)	231 (19.2)	
120,000–500,000	1,054(64.9)	272(64.9)	782(65.0)	
>500,000	249(15.4)	58(13.8)	191(15.9)	

Bold values indicate *P* < 0.05.

**Figure 1:**
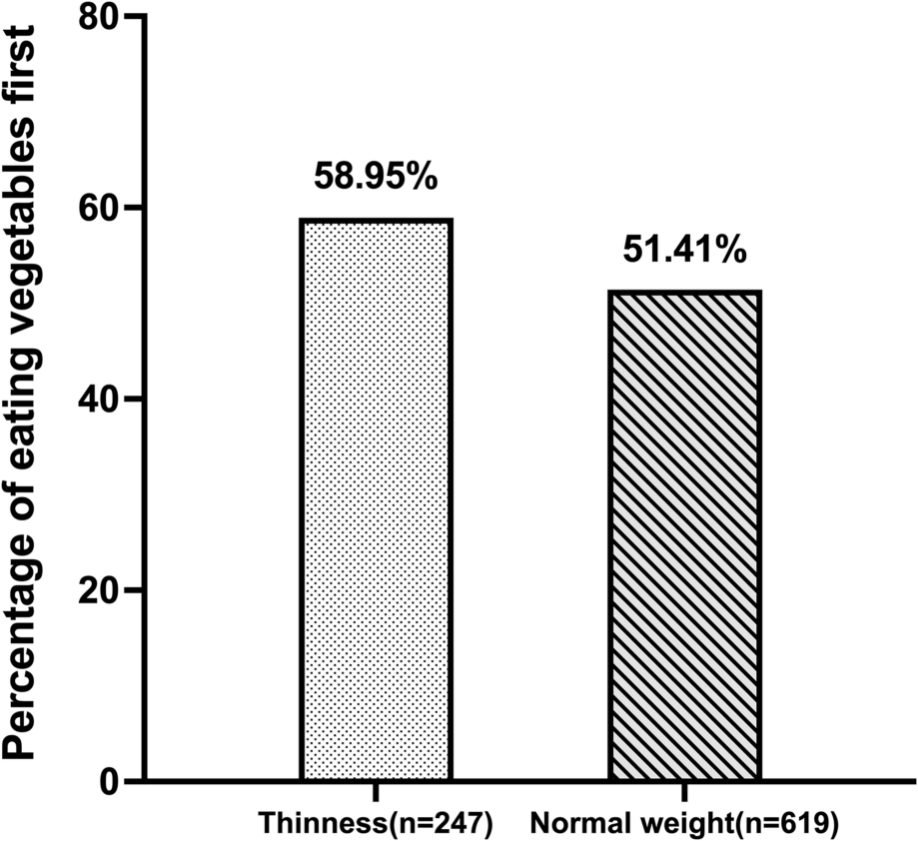
Proportion of children in the thinness and normal weight groups who ate vegetables first.

Overall, 53.4% and 46.6% of the children, respectively, ate vegetables and meat/fish first during meals. Figure 1 shows the differences in order of eating between thinness and normal-weight preschoolers. A higher proportion of children with thinness ate vegetables at the start of a meal as compared children with normal weight (58.95% vs. 51.41%, *P* = 0.008).

After adjusting for sex, children who ate vegetables first were 1.356 times as likely (95% CI: 1.083–1.698, *P* = 0.008) to be thin as compared to children who ate meat/fish first (Model 1, Table 2). After adjusting for mother and father's educational level, the effect of eating vegetables first during a meal on thinness in preschool children remained significant (OR: 1.295, 95% CI: 1.030–1.629, *P* = 0.027) (Model 2, Table 2). Subsequent adjustments to the BMI categories of the mothers and fathers did not completely weaken this association (OR: 1.280, 95% CI: 1.016–1.612, *P* = 0.036) (Model 3, Table 2).

**Table 2:** The effect of eating vegetables first on risk of thinness in preschoolers.

Model	B	Wald *χ*2	*P*	OR	95%CI
Model 1	0.305	7.029	0.008	1.356	1.083–1.698
Model 2	0.259	4.891	0.027	1.295	1.030–1.629
Model 3	0.247	4.382	0.036	1.280	1.016–1.612

Eating meat/fish as reference.

Model 1: Adjust for children's sex.

Model 2: Model 1 + adjust for father's and mother's educational levels.

Model 3: Model 2 + adjust for father's and mother's BMI category.

## 4 Discussion

Currently, much attention is focused on childhood obesity. However, thinness is an important issue that remains to be addressed. The factors associated with childhood thinness are complex and include community, family, socioeconomic, and cultural influences as well as children's feeding practices. Eating habits and food choices affect nutrition and energy expenditure. High consumption of unhealthy foods as well as unhealthy eating habits are risk factors for child mortality, whereas good eating habits are essential for lifelong health (7). Children affected by thinness may consume fewer dietary nutrients, such as vitamins and minerals, which are necessary for growth and development (18).

Some studies have found that childhood thinness is associated with a familial predisposition to low body weight, with an intergenerational transmission of thinness (19). This is consistent with our research, which found that children of mothers with thinness were more likely to be thin. This emphasizes the important role of family in influencing childhood thinness.

### 4.1 Association of order of eating with thinness

Our study found that children who habitually ate vegetables first at meals were more likely to be thin compared with those who ate meat/fish first. To the best of our knowledge, few studies have examined the effects of order of eating on thinness in preschool children. There are several possible explanations for these findings. First, starting a meal by eating low-energy-density foods (vegetables) may better promote satiation and reduce energy intake during the meal compared with eating high-energy-density foods (meat/fish) first (20, 21). Dietary studies on children found that reducing the energy density of food over a 2 day period reduced the cumulative energy intake (22). As the body's accumulated energy decreases, children tend to become leaner.

Vegetables are rich in vitamins, dietary fiber, and trace elements. Dietary fiber has been associated with several potential weight control mechanisms, including reducing dietary energy density, promoting satiation and fullness, reducing intestinal metabolic energy absorption, and regulating the growth of intestinal flora to promote a thinness body weight status (10, 11). Most vegetables are considered to have low energy density, glycemic load, and dietary fat. All these properties promote a negative balance of energy formation and reduce the chances of weight gain in the long-term (12, 23). In addition, vegetables are rich in phytochemicals that may play an important role in lipid energy metabolism, adipose tissue growth and differentiation, and adipocyte apoptosis (24, 25).

### 4.2 Reasonable eating habits and diet education

This study showed that the order in which food is eaten at a meal affects the risk of thinness in children. Strong evidence suggests that children's eating behaviors are overwhelmingly formed during the preschool years (26). Early interventions to develop healthy eating habits at this age can continue into adulthood. As children grow, their energy requirements also increase and they require adequate food intake to maintain an age-appropriate weight. Therefore, it is vital that they obtain their energy from a varied, healthy, and balanced diet. The inability of children to meet their increasing energy and nutritional needs leads to childhood thinness. In our study, children who consumed vegetables first had a significantly increased risk of being affected by thinness. Therefore, eating meat/fish first at mealtimes may be a better choice for children affected by thinness. We believe the findings of our study can serve as dietary references for families and schools.

### 4.3 Public health implications

The study revealed a 28% higher risk of children with thinness who initiated meals with vegetables compared to those who consumed meat/fish first (adjusted OR=1.280, 95% CI: 1.016–1.612). The findings carry important public health implications. First, it identifies a low-cost, easily implementable target for nutritional intervention. Adjusting consumption sequence (prioritizing meat/fish vs. vegetables) serves as a feasible micro-habit modification that requires no changes to food types or additional economic burden, making it suitable for implementation in households and childcare settings. Second, it provides evidence-based guidance for precision nutrition in children with thinness. The study is the first to reveal that “eating vegetables first” may increase thinness risk, suggesting that children with thinness should prioritize energy-dense foods (meat/fish). This challenges the conventional one-size-fits-all recommendation of “vegetables first”. Third, it advances the theoretical framework for child nutrition interventions by advocating for the integration of “dietary behavior dynamics”(e.g., food consumption sequence) into pediatric nutritional assessment systems. This addresses a critical gap in current guidelines that primarily focus on food types and portions. In conclusion, this research introduces a novel behavioral fine-tuning approach to childhood malnutrition prevention, shifting public health strategies from “what to eat” to “how to eat”. It holds promise as a cost-effective strategy for improving child nutrition in resource-limited regions.

### 4.4 Strengths and limitations

Few studies have examined the correlation between order of eating and health in children. This study provides new evidence on the relationship between order of eating and body weight in Chinese children. However, this study had several limitations. First, it was conducted in a city in coastal China; hence, the study population is not representative of the entire population of the country. Second, as a cross-sectional study, this analysis is limited to associations rather than causation. Third, data on weight and height were self-reported by the children's parents; thus, recall bias may have affected the accuracy of the data. However, there is evidence that self-reported height and weight are highly correlated with measurements that can be used to estimate BMI status in population epidemiological surveys (27). Fourth, unmeasured confounders (e.g., total energy intake, physical activity, sleep patterns, and dietary habits) create potential residual confounding. Fifth, we did not examine the relationship between distinct growth indicators: thinness (low BMI-for-age), stunting (low height-for-age), underweight (low weight-for-age), and wasting (low weight-for-height), which should be further explored in the future. Sixth, we were unable to provide consistency and stability regarding the order of eating each meal. Seventh, we used only one question to measure eating vegetables or meat/fish first at the start of a meal, which may introduce misclassification and lacks granularity and lead to recall and social desirability bias. We did not use food frequency or dietary recall instrument in this study. We did not examine the effects of food quantity and eating other foods first, such as dessert, rice, or soup. In addition, cultural variations in meal composition and portion sizes and unaccounted macronutrient intake may affect measurement accuracy.

## 5 Conclusion

Our findings revealed that the food consumed first during meals was related to the children's weight status. For children with thinness, we do not recommend that they eat vegetables first, but rather encourage them to eat meat/fish first.

## Data Availability

The data analyzed in this study is subject to the following licenses/restrictions: The date that support the findings of this study are not publicly available due to their containing information that could compromise the privacy of research participants but are available from the corresponding author. Requests to access these datasets should be directed to Meixian Zhang, zhangmx5935@enzemed.com.
